# Measurement Tools in Occupational Therapy Practice Primary School Setting: A Scoping Review

**DOI:** 10.1155/oti/2679849

**Published:** 2025-12-03

**Authors:** Putri Ashari, Megumi Shiraishi, Yu Ishibashi

**Affiliations:** ^1^Department of Occupational Therapy, Tokyo Metropolitan University, Hachioji, Tokyo, Japan; ^2^Department of Occupational Therapy, Kyorin University, Mitaka, Tokyo, Japan

**Keywords:** measurement, primary school, school-based occupational therapy

## Abstract

**Introduction:**

Occupational therapists play a crucial role in supporting students' participation in primary school by assessing factors that influence their performance. Measurement tools are essential for identifying supports and barriers to participation; however, their alignment with the International Classification of Functioning, Disability, and Health—Child and Youth Version remains unclear.

**Objective:**

This scoping review was aimed at identifying available measurement tools used in occupational therapy practice in the primary school setting and mapping their focus according to the International Classification of Functioning, Disability, and Health—Child and Youth Version components.

**Methods:**

A comprehensive scoping review was conducted following the Joanna Briggs Institute Manual for Evidence Synthesis across four databases (PubMed, CINAHL, MEDLINE, and Web of Science), supplemented by a targeted gray literature search from ProQuest, and hand-searching articles between 2007 and 2025. Eligible studies included quantitative, qualitative, and mixed-method research that utilized measurement tools by OT in primary school settings. A total of 705 articles were retrieved, with 41 meeting the inclusion criteria.

**Results:**

Fifty-two measurement tools used by school-based occupational therapy were identified. Although schools encompass various occupations such as self-care tasks, learning activities, school leisure activities, and social activities, this literature review found that school-based occupational therapy practice in primary school settings primarily addressed handwriting as a student occupational problem. The most frequently used tools were the Minnesota Handwriting Assessment, the Beery-Buktenica Developmental Test of Visual-Motor Integration, the Persian Handwriting Assessment Tool, and the Evaluation Tool for Children's Handwriting. At the same time, most tools focused on handwriting assessment, and only a few incorporated environmental factors.

**Conclusion:**

These findings highlight the predominant focus of school-based occupational therapy measurements on cognitive and motor functions, with limited attention to environmental factors. Future research considers prioritizing the development of more comprehensive tools that holistically assess students' occupational performance.

## 1. Introduction

Children in school settings have a role as students that obligates them to perform and participate in several school occupations [1, 2]. However, many students are being restricted from participating in school occupations they need to perform due to dynamic interactions between personal factors, environmental factors, and school occupations themselves [3]. School-based occupational therapists support students in engaging in meaningful and essential occupations related to access, learning, and participation in school [4]. As an initial step, occupational therapists must identify aspects that caused students' occupational or participation dysfunction [4]. Therefore, occupational therapists use measurement tools to identify supports and barriers to students' participation in school activities [5]. Understanding the focus of these tools is essential to ensuring comprehensive and evidence-based occupational therapy services in educational environments.

Since schools are inherently interprofessional environments, occupational therapists must conduct a dynamic performance analysis to create a comprehensive and collaborative program [1]. The International Classification of Functioning, Disability, and Health—Child and Youth Version (ICF-CY) has proven to be an effective framework for occupational therapists and other professionals in conceptualizing and categorizing children's functioning. This framework provides components that clarify the dynamic interaction between health conditions and contextual factors [6, 7]. It offers a common language for describing health and disability, organized into four core components of the biopsychosocial model: body functions, body structures, activity and participation, and environmental factors [8]. Crucially, the ICF-CY shifts the focus from an individual's deficits to their participation in typical age- and grade-level occupations, reflecting a holistic understanding of student functioning, enabling occupational therapists to deliver services beyond simple remediation to include necessary accommodations and environmental modifications, reflecting the distinct nature of school-based services compared to traditional medical settings [9, 10]. By aligning measurement tools with the ICF-CY framework, it becomes possible to assess whether existing evaluations comprehensively address all key aspects of child engagement and participation, particularly in the context of school-based occupational therapy.

Despite the availability of various measurement tools for occupational therapy practice, evidence regarding their alignment with the ICF-CY framework remains limited. Few review studies have been conducted to examine measurement tools for students in primary or elementary school [11–13]. Notably, none of these studies explicitly used the ICF-CY component as the standard for analyzing the focus of each available measurement tool. Among these studies, Nakajima et al. [11] explored the measurement tools used by occupational therapy in special needs school settings in Japan. Meanwhile, two of them are scoping reviews related to school engagement measurement [13] and the measurement of self-regulation in elementary children [12].

Addressing the existing knowledge gap in current literature, this scoping review was aimed at identifying available measurement tools used in occupational therapy practice in the primary school setting and mapping their focus according to the International Classification of Functioning, Disability, and Health (ICF) components. A scoping review serves as a valuable approach for identifying and mapping the available evidence related to specific objectives and review questions regarding key concepts [14–16]. Accordingly, this review formulated the research question as follows: “What measurement tools are used by occupational therapists practice in primary school settings? And how is the mapping of each measurement tool according to the ICF components?” Adopting the PCC acronyms, the population of this review includes students or children in primary or elementary school, the concept refers to occupational therapy measurement tools, and the context encompasses school-based or school settings. The findings of this review may contribute to supporting evidence-based occupational therapy practice in primary school settings by providing a comprehensive overview of available measurement tools and their alignment with the ICF-CY framework.

## 2. Methods

This scoping review was conducted following the procedures outlined in the Joanna Briggs Institute (JBI) Manual for Evidence Synthesis [17]. A scoping review is essential for exploring the breadth of the literature, mapping and summarizing the evidence, and guiding future research [17, 18]. For reporting, this review adhered to the Preferred Reporting Items for Systematic Reviews and Meta-Analyses Extension for Scoping Reviews (PRISMA-ScR) guidelines [19].

### 2.1. Eligibility Criteria

Since the ICF-CY was published in 2007, this scoping review includes articles in English between 2007 and 2025, involving articles conducted by occupational therapists. Quantitative, qualitative, and mixed-method research designs were included to ensure a comprehensive exploration of the measurement tools that may be available and commonly utilized by occupational therapists in primary school settings. Participants in the included articles are primary school students aged 5–12, as defined in the International Standard Classification of Education (ISCED 2011) [20].

### 2.2. Search Strategy

**Figure 1:**
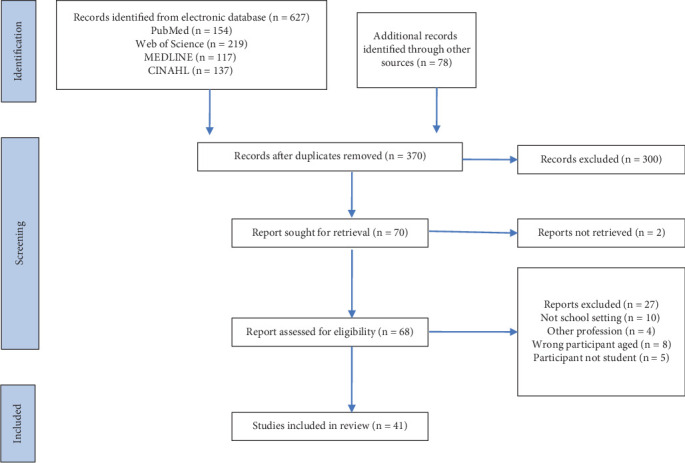
Flow chart of the selection process.

This scoping review identifies potentially relevant literature from four electronic databases: PubMed, CINAHL, MEDLINE, and Web of Science, which were systematically searched multiple times between July 2024 and October 2025, with the final search completed on October 1, 2025. These databases were selected as primary resources of medical, rehabilitation, and educational literature. Supplementary hand searches were conducted up to 10 pages of Google Scholar, through the reference lists of previous studies, and in ProQuest Dissertations and Theses Global for the gray literature. The systematic research was conducted across all databases using the following Boolean terms: (student∗ OR child∗) AND (elementary OR primary) AND (assess∗ OR measur∗ OR evaluat∗) AND (“occupational therap∗” OR OT) AND (school OR education). The results of the final systematic search are illustrated in Figure 1 (identification).

### 2.3. Study Selection

The findings from each database search and hand-search were imported into Rayyan as data management for deduplication and screening tasks [21]. After removing duplicate articles, three members of the research team independently screened titles and abstracts (*n* = 370). Based on the eligibility criteria, 300 articles were determined to be irrelevant and excluded. The exclusion criteria are literature review articles, studies conducted by another profession, and studies not set in a school environment. A total of 41 articles were eligible and included in the scoping review. Conflicts during the screening process were solved through consensus by at least two of the three authors.

### 2.4. Charting the Data

This scoping review modified the data charting form developed by Arksey and O'Malley [22] and employed a descriptive–analytic approach to extract relevant data from each article. In total, 41 articles were examined in detail, and data extraction was conducted accordingly.

### 2.5. Summarizing and Reporting the Data

The data were summarized by descriptive and analytical thematic approaches, as previously applied in studies with a similar purpose [23]. The descriptive summary contains information on the author, study location, study design, objective, participants, measurement tools, and intervention. Additionally, available measurement tools were classified based on the developer's profession, country of origin, purpose, instrument type, and reported validity and reliability.

For thematic analysis, each measurement, based on its focus, was linked to the ICF component by the linking process. The linking process is an approach to associating health information with the ICF reference framework to ensure comparability across different sources [24]. To improve clarity and consistency, the linking process was restricted to first-level ICF categories rather than more detailed classifications, as follows from the previous review study with the same methodology [23]. Each measurement is categorized based on its focus on the four ICF components: body function (b) if it assessed student physiological functions of body systems as well as psychological functions, body structure (s) as it assessed anatomical parts of the body, activities and participation (d) for evaluating problems that students may experience in involvement in life situations, and environmental factors (e) including assessments related to the physical, social and attitudinal environment. This classification component was coded with the letters b, s, d, and e, followed by a numeric code of the chapter number. For example, the component “body function” contains the following code: b2-sensory functions and pain (see Table S1). The agreement between all researchers was achieved for all measurement linking procedures.

## 3. Results

### 3.1. Characteristics of Studies

A total of 705 articles were identified in the initial search using predefined keywords and the hand-search method. After the deduplication, screening, and application of the exclusion criteria, 41 articles met the inclusion criteria for this scoping review, as shown in Figure 1 (screening). These studies were primarily conducted in North America and Asia, with other studies conducted in Europe, Africa, Australia, and South America. Based on the study design, 15 studies used nonrandomized controlled trials. The remaining studies included psychometric studies, observational studies, mixed-method studies, crossover studies, randomized controlled trials, comparative studies, case studies, and correlative studies as study designs. The participants in the studies comprised both typical students and students with diverse conditions. Almost half of the studies do not provide intervention (*n* = 19) to the participants, whereas 13 studies provide handwriting intervention within various approaches and programs. Detailed information about the authors, the year of publication, the study location, the objective, participants, measurement, and intervention in each article is presented in Table 1.

### 3.2. Characteristics of Measurement

There are 52 measurements identified from 41 articles. Those measurements were dominantly developed in the United States. Most measurements are norm-referenced (57.7%) and criterion-referenced (30.8%). In terms of the administration procedure, performance-based (42.3%) measurement is a common administration procedure in primary school settings. The measurements available for students are frequently employed for screening and documenting a child's status (36.5%) and documenting a child's status in conjunction with planning intervention (17.3%). The Minnesota Handwriting Assessment (MHA), Beery-Buktenica Developmental Test of Visual-Motor Integration (VMI), Persian Handwriting Assessment Tool (PHAT), and Evaluation Tool for Children's Handwriting (ETCH) were the most frequently used tools. Although numerous measurement tools exist, not all the articles in this review provided information on the psychometric properties of each measurement. Detailed information about measurement tools is shown in Table 2.

### 3.3. Outcome Measures and ICF Components Linking

Based on the ICF classification, none of the identified measurement tools were focused on body structures; instead, they primarily related to body function, activities, and participation, or environmental factors. Thirty-four measurements are classified as multicomponent as they cover two or three components of the ICF classification. The summary of measurement tools by ICF classification is presented in Table 3.

Thirty-eight measurements assessed body function focused on the ICF chapters b1, b2, b4, b7, and b8, with the majority focused on mental functions (b1). Almost all the measurements are related to activities and participation, particularly in the components of learning and applying knowledge (d1), mobility (d4), and interpersonal interactions and relationships (d7). These tools primarily assess functional task performance central to the student role. Additional measurements relate to general tasks and demands (d2), communication (d3), self-care (d5), domestic life (d6), major life areas (d8), and community, social, and civic life (d9). This scoping review found that only four measurements considered environmental factors, including support and relationships (e3) and attitudes (e4). It suggests a significant conceptual gap, potentially limiting the comprehensive dynamic performance analysis as recommended by the biopsychosocial model based on ICF.

## 4. Discussion

This scoping review is intended to identify available measurement tools for occupational therapists' practice in primary school settings and map these tools according to the ICF-CY components. A total of 41 articles were incorporated, comprising 52 available measurement tools. It also captures various occupational therapy intervention services in primary school settings that align with our domain practices. Although pediatric occupational therapists are likely familiar with the available measurements, this review provides a comprehensive reference for occupational therapists working in a primary school setting. It enables them to select the most effective measurements to optimize their intervention planning. Mapping measurement through the ICF-CY framework not only facilitates interdisciplinary collaboration between occupational therapists and school professionals but also enhances occupational therapists' ability to evaluate person–environment interactions that impact student participation. Moreover, the ICF-CY classification gives a standardized structured approach that enables teachers to see the student's situation more clearly, fostering a shared framework for education and intervention [114, 115].

Fifty-two measurement tools were identified, with 38 tools assessing body function, primarily addressing ICF chapters b1, b2, b4, b7, and b8, with a predominant focus on mental functions (b1). Additionally, 29 tools examined activities and participation components related to learning and applying knowledge (d1), while 24 tools assessed mobility (d4). Specific mental functions, including attention, memory, psychomotor skills, basic cognitive functions, language function, calculation, and sequencing complex movements, play a crucial role in successful academic performance and participation in school-related activities. Since school activities are inherently tied to the educational task, students are expected to learn and apply knowledge (d1) through purposeful sensory experiences (e.g., watching, listening, and touching) and basic learning such as copying, reading, writing, calculating, and problem-solving. Likewise, mobility (d4) contributes significantly to school performance, as students need to maintain proper sitting posture for extended periods and develop fine motor skills that are necessary for writing and other academic tasks. These core components are fundamental for students to perform successfully in school, emphasizing the need for occupational therapists to critically evaluate whether the measurement tools that they use in practice have effectively captured both underlying capacities and real situation performances.

This review identified the MHA, Beery-Buktenica Developmental Test of VMI, PHAT, and ETCH as the most frequently used measurements. The primary focus of these measurements is to evaluate student body function (b1 and b2) along with activities and participation (d1 and d4) related to handwriting activities by emphasizing fine motor skills and VMI. Developed in the early 1990s, the MHA is widely used by occupational therapists to assess and measure treatment effectiveness for students with handwriting difficulties [67]. It effectively measures writing legibility and speed in near-point copying, demonstrating strong construct validity and reliability as supported by multiple previous studies [116–118]. The PHAT was the first standardized Persian handwriting tool designed to assess legibility and speed in near-point copying and dictation for second and third-grade primary school students among Persian-speaking students [68]. As a relatively recent tool, it has demonstrated high validity based on exploratory factor analysis and confirmatory factor analysis, along with high internal consistency reliability (copying: *α* = 0.72–0.99, dictation: *α* = 0.74–0.99) [77]. The VMI is widely used in occupational therapy practice to assess VMI skills essential for handwriting development [119]. It has demonstrated excellent validity, reliability, and internal consistency, with no significant differences across gender, ethnicity, socioeconomic status, or residency [120]. However, as a screening tool, the VMI may lack the sensitivity to detect handwriting performance improvements resulting from occupational therapy interventions [121, 122]. The other measurement that focuses on the readability of letters, words, and numbers at a glance and out of context is the ETCH [123]. However, it has low interrater reliability and moderate validity coefficients [124, 125].

While these handwriting measurement tools have strong psychometric properties, their relevance is limited by the current situation, including curriculum shifts and increased digital accessibility in schools. Recent studies [126, 127] mentioned factors influencing handwriting challenges, including language difficulties, letter knowledge, executive function, and writing surface, that were not adequately captured by these measurement tools. Similarly, Weintraub [128] mentions it is important for occupational therapists to be aware that handwriting performance is limited or facilitated by various personal factors (including age, gender, cognitive and psychological, sensory–perceptual, motor functions, pencil grip, and hand preference) and environmental context factors (including ergonomic factors, writing utensil type, and linguistic context) as aligned with the ICF. This review captures that all the measurement tools used to evaluate handwriting performance (ETCH, MHA, PHAT, The Print Tool, Here's How I Write: A Child's Self-Assessment, BHK Concise Assessment Method for Children's Handwriting, Arabic Handwriting Assessment, Arabic Keyboarding Assessment, Hebrew Keyboarding Assessment, Handwriting Proficiency Screening Questionnaire for Children, Handwriting Legibility Scale, and Chinese Handwriting Analysis System) focus on personal factors (body function and activities and participation).

Thirty-four measurements are classified as multicomponent, covering two or three components of the ICF classification. Among these, there are two measurement tools, School Function Assessment (SFA) and Short Child Occupational Profile (SCOPE), which emerge as comprehensive tools that offer a holistic perspective on student school performance functioning that integrates body function (b), activity and participation (d), and environmental factors (e). Specifically, both tools share the same focused component on mental and executive function (b1), basic and complex interpersonal interactions (d7), school education (d8), support and relationship (e3), and attitudes (e4). Unlike other standardized measurements that primarily identify delays and deficits, the SCOPE with good construct validation is designed to detect occupational strengths and challenges through various factors such as volition, habituation, communication/interaction skills, process skills, motor skills, and environment [129–131]. It considers broader occupational engagement in the school context, not limited to specific components. Similarly, the SFA, as a standardized measurement that demonstrates strong validity and reliability, not only assesses participation in primary school-based tasks but also provides targeted intervention planning by identifying difficulty areas and synchronizing therapy goals with educational priorities, which can be used together with school personnel [132, 133]. Since both of these tools were developed by occupational therapists, they emphasize their alignment with occupational therapy principles and perspectives. This highlights the profession's role in fostering school participation, not limited to addressing skill development [1]. By bridging the gap between skill-based assessment and broader occupation-centered evaluations, these tools contribute to the effectiveness of school-based occupational therapy that promotes meaningful intervention for students.

The findings of this scoping review indicate that the available measurement tools in school-based occupational therapy practice employ a diverse range of methods to assess students, including performance-based assessment, observation-based evaluations, semistructured interviews, self-report questionnaires, and proxy-report questionnaires administered to teachers and parents. The majority of the available measurements are performance-based, such as the Dynamic Occupational Therapy Cognitive Assessment for Children (DOTCA-Ch), the Motor Assessment Battery for Children (MABC), and the Primary Spelling Inventory (PSI). However, performance-based measurement tools give us opportunities to assess the capacity of what a person can do under standardized and optimal conditions, but not what they can actually do in their habitual environment [134]. Therefore, these measurement tools may fail to accurately assess students' functional performance, as contextual factors significantly influence their participation. This limitation raises concerns about the relevance of the results of these measurement tools in natural settings and questions their applicability in school-based occupational therapy practice.

Effective collaboration with school personnel will support school-based occupational therapists in delivering comprehensive interventions. Given the necessity for occupational therapists to collaborate with school personnel, the use of proxy measures, particularly those obtained from teachers, such as Teacher Skillstreaming Checklists and Sensory Profile Questionnaires (SPQs), can help bridge the gap between occupation-based practice and school priorities [1]. Additionally, these tools assist occupational therapists in understanding children in the real-world school context. For instance, collaborating with teachers allows occupational therapists to gain a deeper understanding of children's sensory needs in the classroom setting [135]. Since we are in an interprofessional environment, implementing interprofessional assessments is essential for occupational therapists to provide contextually relevant and effective practice in school settings [1]. Furthermore, it enhances the connectivity between therapeutic intervention and educational goals.

### 4.1. Limitations

This scoping review has some limitations. The review followed specific inclusion and exclusion criteria, which may have restricted the range of studies identified. For example, first, this review was limited to articles written in English and published after 2007. However, this criterion was established to ensure that the included articles were published after the ICF Children and Youth version was published, based on the assumption that more comprehensive measurements may have been developed in subsequent years. Second, this review selected only articles that were written in English, which might have missed articles in other languages. Yet, this criterion was chosen due to limited resources for translation and interpretation, as well as the dominance of English in academic communication, which ensures the inclusion of high-impact and frequently cited research.

## 5. Implications for Occupational Therapy Practice

• School-based occupational therapists require appropriate measurement tools to guide their practice effectively. The findings of this review have been collected and mapped to the relevant ICF-CY components for all identified measurements that can be used in primary school settings, as shown in Table 2.
• When selecting measurement tools, school-based occupational therapists should carefully consider the specific purpose and intended application of each measurement tool to ensure its suitability for assessment and intervention planning.
• There remains a limited number of comprehensive measurements that include environmental factors, suggesting a need for further research and development in this area. Additionally, widely available measurements focus on handwriting; further research is warranted to develop measurement tools that address other critical school tasks.

## 6. Conclusions

This scoping review was aimed at capturing a wide range of available measurements that have been used by occupational therapists since 2007 in primary or elementary school settings and mapping each measurement tool to the ICF-CY components. Findings from 13 articles discovered that occupational therapy practice in primary school settings primarily focuses on handwriting assessment and intervention. The most frequently used measurement tools were MHA, VMI, PHAT, and ETCH. These tools are performance-based, norm-referenced, and criterion-referenced measurements commonly used for screening, planning intervention, and documenting intervention progress. However, these measurements predominantly assess activities and participation within the ICF-CY component. This pattern appears consistent with 39 other measurements, which also emphasize activities and participation. Only three measurements explicitly incorporated environmental factors, and among them, only two measurements were developed specifically by and for occupational therapy. These findings suggest a need for the development of more comprehensive measurement tools that consider environmental factors alongside body function and activity participation. Expanding measurement tools in this manner might support students' full participation in educational settings.

## Figures and Tables

**Table 1:** Characteristics of included studies.

**No.**	**Author(s), year of publication**	**Study location**	**Study design**	**Objective**	**Participants**	**Measurement**	**Intervention**
1.	Ratzon et al., 2007 [25]	Israel, Asia	Randomized experimental design	To test the efficacy of a short-term treatment on the fine-motor and graphomotor skills	Student with low visual–motor integration skill (*n* = 52)	1. Beery-Buktenica Developmental Test of Visual-Motor Integration (VMI) [26] 2. Developmental Test of Visual Perception-2 (DTVP-2) [27] 3. Bruininks-Oseretsky Test of Motor Proficiency [28]	Graphomotor intervention included 12 sessions, each held once a week for 45 min, using various fine motor activities

2.	Pfeiffer et al., 2008 [29]	Italy, Europe	Randomized experimental design	To investigate the effectiveness of a type of dynamic seating system, the Disc ‘O' Sit cushion, for improving attention to task among students with attention difficulties	Second-grade student with attention difficulties (*n* = 63)	1. Behavior Rating Inventory of Executive Functioning (BRIEF) [30]	Disc ‘O' Sit cushion to place on his or her regular classroom seat for 2 h a day for 2 weeks

3.	Wight and Chappro, 2008 [31]	Sydney, Australia	Observational design	To investigate teacher perception of the social competence of primary-school male children with learning difficulties in the classroom context	• Male student with learning disability (*n* = 21) • Comparison group typical student (*n* = 21)	1. Teacher Skillstreaming Checklist [32]	No intervention

4.	Egilson and Traustadottir, 2009 [33]	Iceland, Europe	Mixed-methods design	To investigate the factors that facilitate or hinder school participation of students with physical disabilities and to explore the interaction of those factors	• Qualitative phase: 14 students with physical disabilities (defined as key participants), 17 parents, and 18 teachers • Qualitative phase: Students with primary physical impairment (*n* = 32 students)	1. School Function Assessment (SFA) [34]	No intervention

5.	Zwicker and Hadwin, 2009 [35]	Victoria and Vancouver, North America	Randomized experimental design	To compare the effectiveness of cognitive vs. multisensory interventions in improving handwriting legibility of students in first and second grade	Student with handwriting difficulties (*n* = 72)	1. Evaluation Tool for Children's Handwriting (ETCH) [36]. 2. Beery-Buktenica Developmental Test of Visual-Motor Integration (VMI) [26].	Handwriting intervention based on: • Cognitive intervention • Multisensory intervention

6.	Mackay et al., 2010 [37]	Sydney, Australia	Nonrandomized experimental design	To determine the feasibility and outcomes of the Log Handwriting Program	Student with handwriting difficulties (*n* = 16)	1. Minnesota Handwriting Assessment (MHA) [38]	Handwriting training using the Log Handwriting Program based on task-specific training

7.	Richmond and Holland, 2010 [39]	South Africa, Africa	Correlative design	To find the relationship between the teachers' classroom observations and visual perceptual difficulties	Students with at least one learning-related task difficulty, such as reading, writing, spelling, or mathematics (*n* = 206)	1. Developmental Test of Visual Perception-2 (DTVP-2) [27] 2. Test of Visual Perceptual Skills (nonmotor) Revised (TVPS-R) [40] 3. Jordan Left-Right Reversal Test (JLRRT) [41]	No intervention

8.	Case-Smith et al., 2011 [42]	Columbus, United States	Nonrandomized experimental design	To evaluate the effects of handwriting and a writing program that was co-taught by teachers and an occupational therapist	First-grade student (*n* = 19)	1. Evaluation Tool for Children's Handwriting (ETCH) [36] 2. Minnesota Handwriting Assessment (MHA) [38] 3. Woodcock–Johnson III Tests of Achievement (WJIII) [43]	Coteaching handwriting program collaboration of occupational therapist and classroom teacher involved 45-min sessions implemented twice a week for 12 weeks (24 sessions total)

9.	Case-Smith et al., 2012 [44]	Columbus, United States	Nonrandomized experimental design	To evaluate the effects of handwriting and a writing program that was co-taught by teachers and an occupational therapist	First-grade student (*n* = 36)	1. Evaluation Tool for Children's Handwriting (ETCH) [36] 2. Woodcock–Johnson III Tests of Achievement (WJIII) [43]	Coteaching handwriting program collaboration of an occupational therapist, a classroom teacher, and an intervention specialist involved 45-min sessions implemented twice a week for 12 weeks (24 sessions total)

10.	Howe et al., 2013 [45]	New York, North America	Nonrandomized experimental design	To examine the effectiveness of two approaches used in elementary schools to improve children's handwriting	Student with handwriting difficulties (*n* = 72)	1. Minnesota Handwriting Assessment (MHA) [38] 2. Beery-Buktenica Developmental Test of Visual-Motor Integration (VMI) [26]	Handwriting intervention based on: 1. Motor learning approach 2. Visual perceptual motor approach

11.	Garg et al., 2013 [46]	New York, North America	Nonrandomized experimental design	To examine the effectiveness of the Get Ready to Learn (GRTL) program in improving classroom behaviors of elementary students with disabilities	Students diagnosed with multiple handicapping conditions, developmental disabilities, or ASD (*n* = 51)	1. Get Ready to Learn Supplemental Data Sheet [47]	Intervention to reduce maladaptive behavior using the Get Ready to Learn program based on the preparatory method

12.	Lau et al., 2013 [48]	Nevada, North America	Nonrandomized experimental design	To investigate the effectiveness of an occupation-based after-school program to promote healthy lifestyle choices for children at risk	Students who attend the after-school program (*n* = 27)	1. Child and Adolescent Trial for Cardiovascular Health: Health Behavior Questionnaires (HBQ) [49] 2. The Physical Activity Questionnaire (elementary school) [50]	After-school programs using occupation-based approaches focus on empowering children to make choices within their daily routine and to develop long-term healthy habits

13.	Li-Tsang et al., 2013 [51]	Hong Kong, Asia	Psychometric research design	To validate the Chinese Handwriting Analysis System (CHAS), which is designed to measure both the process and production of handwriting	Student Grades 1–6 (*n* = 734)	1. Chinese Handwriting Assessment System (CHAS) [51]	No intervention

14.	Hape et al., 2014 [52]	Indianapolis, North America	Nonrandomized experimental design	To determine the effectiveness of Handwriting Without Tears	Typical student (*n* = 43)	1. The Print Tool [53]	Handwriting intervention based on: 1. Multisensory approach 2. Teacher's common writing program

15.	Krishnaswamy and Srimathveeravalli, 2014 [54]	New York, North America	Randomized experimental design	To investigate the effects of a robot-mediated visual–motor program on improving the visual–motor skills of children with learning disabilities and visual–motor delays	Students with a learning disability and visual–motor delay (*n* = 25)	2. Beery-Buktenica Developmental Test of Visual-Motor Integration (VMI) [26]	Visual–motor integration intervention using: 1. Visual–motor activities 2. The Novint Falcon haptic interface

16.	Roberts et al., 2014 [55]	Canada, North America	Cross-over design	To determine the effectiveness of Handwriting Without Tears (HWT) on students' handwriting and perception of skills	Typical student (*n* = 149)	1. Minnesota Handwriting Assessment (MHA) [38] 2. Canadian Occupational Performance Measure (COPM) [56]	Handwriting intervention based on: • Multisensory approach • Teacher's common writing program

17.	Almomani et al., 2014 [57]	Jordan, Asia	Observational design	To investigate cognitive functioning among elementary school children in Jordan	Typical student (*n* = 468)	1. Loewenstein Occupational Therapy Cognitive Assessment (LOTCA) [58]	No intervention

18.	Cermak and Bissell, 2014 [59]	California, North America	Psychometric research design	To examine the content and construct validity of Here's How I Write: A Child's Self-Assessment	• Students with handwriting difficulties (*n* = 20) • Student without handwriting problem (*n* = 20)	1. Here's How I Write (HHIW) [60]	No intervention

19.	Pearton et al., 2014 [61]	Western Cape, South Africa	Comparative study	To investigate the differences in playfulness of children with and without prenatal alcohol exposure (PAE)	• Students who had positive histories of PAE (*n* = 15) • Typically developing student with no history of PAE (*n* = 15)	1. Test of Playfulness (ToP) [62]	No intervention

20.	Jordan et al., 2016 [63]	Switzerland, Europe	Nonrandomized experimental design	To analyze the efficacy of a program that combines fine motor activities, animated models, exercises on a digital tablet, and paper–pencil exercises	Typical student (*n* = 30)	1. BHK, Concise Assessment Method for Children's Handwriting [64]	Handwriting intervention based on: • Fine motor activities, animated models, exercises on a digital tablet, and paper–pencil exercises • Teacher's common writing program

21.	Saleem and Gillen, 2019 [65]	New York, North America	Nonrandomized experimental design	To examine the effectiveness of mental practice (MP) combined with repetitive task practice (RTP) to rehabilitate handwriting in children	Students in first and second grade who have handwriting difficulties (*n* = 20)	1. The Kids' Imaging Ability Questionnaire (KIAQ) [66]. 2. The Minnesota Handwriting Assessment (MHA) [67]	Handwriting intervention with mental practice (MP) combined with repetitive task practice (RTP)

22.	Havaei et al., 2016[68]	Iran, Asia	Psychometric research design	To develop and validate the Persian handwriting assessment tool (PHAT) for primary school students	Typical student (*n* = 131)	1. Persian handwriting assessment tool (PHAT) [68]	No intervention

23.	Nobahar et al., 2018 [69]	Iran, Asia	Psychometric research design	To develop a tool to assess the performance skills of Iranian children aged 5–7 years and to evaluate their school competency based on the occupational therapy practice framework	Typical student (*n* = 400)	1. School Interim Competency of Performance Skill Battery Scale (SICPSBS) Nobahar et al., 2018 [69]	No intervention

24.	Demarchi et al., 2019 [70]	Brazil, South America	Psychometric research design	To analyze the internal consistency of the Portuguese version of The Dynamic Occupational Therapy Cognitive Assessment for Children (DOTCA-Ch)	Typical student (*n* = 90)	1. The Dynamic Occupational Therapy Cognitive Assessment for Children (DOTCA-Ch) [71]	No intervention

25.	Challita et al., 2019 [72]	Sydney, Australia	Cross-over design	To investigate the impact of a playground social skills program based on the Perceive, Recall, Plan, and Perform intervention	The student with playground interaction participation difficulty (*n* = 16)	1. Goal Attainment Scaling Scores (GAS) [73] 2. The Perceive, Recall, Plan, and Perform (PRPP) [74]	Playground interaction focuses on transitioning social skills intervention by: • Cognitive strategies based on Perceive, Recall, Plan and Perform (PRPP) • Normal playground interaction

26.	Lee and Lape, 2019 [75]	Columbia, South America	Nonrandomized experimental design	To determine the effectiveness of the cognitive approach to improve handwriting legibility and students' perceptions of self-monitoring strategies based on self-monitoring strategies with teacher and occupational therapist collaboration	• Student with IEP (*n* = 5) • Typical student (*n* = 14)	1. Minnesota Handwriting Assessment (MHA) [38]	Handwriting intervention with the Size Matters Handwriting Program focuses on letter sizes and self-monitoring methods by students

27.	Montgomery and Zwicker, 2019 [76]	Vancouver, North America	Nonrandomized experimental design	To evaluate the preliminary effectiveness of this program used in a teacher-taught printing club with occupational therapy support	Grades 2 and 3 students with poor legibility (*n* = 11)	1. Minnesota Handwriting Assessment [67]	Handwriting program with a task-specific printing program based on a cognitive approach (rather than a sensorimotor approach)

28.	Meimandi et al., 2020 [77]	Iran, Asia	Psychometric research design	To determine further validation aspects of the Persian Handwriting Assessment Tool (PHAT) in primary school-aged children	Typical student (*n* = 452)	1. Persian Handwriting Assessment Tool (PHAT) [68]	No intervention

29.	Rajaei et al., 2020 [78]	Iran, Asia	Observational design	To investigate the relationship between sensory processing patterns and sleep quality in primary school children	Typical student (*n* = 231)	1. Children's Sleep Habit Questionnaire (CSHQ) [79] 2. Sensory Profile Questionnaire (SPQ) [80]	No intervention

30.	Havaei et al., 2021 [81]	Iran, Asia	Observational design	To gather comprehensive information about handwriting issues for therapists and related disciplines in Iran	Typical student (*n* = 1.262)	1. Persian Handwriting Assessment Tool (PHAT) [68]	No intervention

31.	Romero-Ayuso et al., 2022 [82]	Spain, Europe	Nonrandomized experimental design	To analyze the effect of a self-regulation program at a primary school on the social interactions of neurotypical children and children with special educational needs, from the teachers' and parents' perspectives	• Students neurotypical (*n* = 100) • Students with special educational needs who had a neurodevelopmental or learning disorder (*n* = 7)	1. Peer Social Maturity Scale (PSMAT) [82]	Intervention for self-regulation and social interaction programs based on the concept that regulation involves behavioral, emotional, and cognitive modulation on the theoretical framework of emotional education, which includes five elements: emotional awareness, emotional regulation, emotional autonomy, social competencies, and competencies for life and well-being

32.	Ohl and Schelly, 2021 [83]	Northeastern, North America	Psychometric research design	To estimate the MCID for the Beery VMI battery of tests in children with autism spectrum disorder (ASD)	Student with autism (*n* = 64)	1. Beery-Buktenica Developmental Test of Visual-Motor Integration (VMI) [26]	No intervention

33.	Kim et al., 2021 [84]	Korea, Asia	Psychometric research design	To verify the validity and reliability of the School Function Assessment (SFA) in South Korea	Student with disability (*n* = 103)	1. School Function Assessment (SFA) [34]	No intervention

34.	Whiting et al., 2023[85]	Boston, North America	Nonrandomized experimental design	To examine the effectiveness of sensory integration intervention paired with teacher consultation to improve functional regulation and active participation in school for students with sensory integration and processing differences	Students with sensory problems (*n* = 3)	1. Short Child Occupational Profile (SCOPE) [86] 2. Behavior Assessment System for Children, Third Edition (BASC–3; [87]) 3. Goal Attainment Scaling Scores (GAS) [73]	Sensory integration intervention combined with teacher consult sessions to provide environment modification for classroom activities

35.	Khoury-Shaheen and Weintraub, 2023 [88]	Israel, Asia	Observational design	To examine the relationship between Arabic handwriting and keyboarding performance of typical elementary school students and to investigate whether these two activities share common underlying functions	Typical student (*n* = 35)	1. Colored Progressive Matrices Test (CPM; [89]) 2. Diagnostic Reading Test in Arabic for Elementary School Students, 2nd−6th grade [90] 3. Finger Succession (FS) [91] 4. Motor Assessment Battery for Children 2nd Edition (MABC-2) [92] 5. Children's Color Trails Test (CCTT; [93]) 6. Arabic Handwriting Assessment (A-HAT) [94] 7. Arabic Keyboarding Assessment (A-KBAT) [95]	No intervention

36.	Tallas and Cole, 2023[96]	Utah, North America	Nonrandomized experimental design	To determine the impact of the Handwriting Without Tears curriculum on a first-grade students' ability to spell	Typical student (*n* = 15)	1. Primary Spelling Inventory (PSI) [97]	Handwriting intervention using a multisensory approach

37.	Gahshan-Haddad and Weintraub, 2023 [98]	Israel, Asia	Observational design	To compare elementary school students' keyboarding performance (speed and accuracy) in two tasks: copying and keyboarding to dictation, and to examine the relationship between underlying functions (reading speed, attention shifting, fine motor skills, and kinaesthetic awareness) and keyboarding performance in the two tasks	Typical student (*n* = 57)	1. Colored Progressive Matrices Test (CPM; [89]) 2. Aleph-Taph [99] 3. Children's Color Trails Test (CCTT; [93]) 4. Purdue Pegboard Test (PPT) [100] 5. Finger Succession (FS) [91] 6. Hebrew Keyboarding Assessment (H-KBAT) [101]	No intervention

38.	Jeong, 2024 [102]	Korea, Asia	Case study	To explore the effects of school-based occupational therapy on children's attention, school adaptation, sensory processing, and motor function for children in special classes in elementary schools in Korea	• Student with ASD (*n* = 1) • Student with intellectual disability (*n* = 1)	1. Korea-Child Behavior Checklist (K-CBCL) [103] 2. School Life Adjustment Scale [104] 3. Sensory Processing Assessment Tool for Schools [105] 4. Bruininks-Oseretsky Test of Motor Proficiency 2 Short Form [106]	School-based occupational therapy program to improve school adjustment, including learning activities, school rules, and interactions, by mediating each child's problems

39.	Romero-Ayuso et al., 2024 [107]	Spain, Europe	Psychometric research design	To translate and study the psychometric properties of the Handwriting Proficiency Screening Questionnaire for Children (HPSQ-C) for the Spanish population	Typical student (*n* = 164)	1. Handwriting Proficiency Screening Questionnaire for Children (HPSQ-C) Spanish version [107]	No intervention

40.	Donica et al., 2025 [108]	Southeast, North America	Observational design	To examine performance-based praxis skills of third- and fourth-grade students with and without handwriting difficulties (HDs)	Typical student (*n* = 33)	1. The EASI [109] 2.Handwriting Legibility Scale (HLS) [110]	No intervention

41.	Lucas Molitor and Naber, 2024 [111]	Midwest region, North America	Nonrandomized experimental design	To explore the feasibility of an 8-week after-school program to promote physical activity, social participation, and well-being among rural elementary-age children	Students who attend the after-school program (*n* = 23)	1. Activity Clock [112] 2. Physical and Social Well-Being Ladders [113]	After-school programs that promote physical activity, social participation, and well-being

**Table 2:** Characteristic measurement included.

**No.**	**Measurement tools, in which article**	**Measurement developer, profession**	**Original country**	**Purpose**	**Instrument type**	**Most related ICF chapters**	**Validity and reliability**
1.	Teacher Skillstreaming Checklist [31]	McGinnis and Goldstein [32], psychologists	United States	Designed to assess children with behavior problems in school settings used by teachers, parents, or child raters	• Approach: Criterion-referenced • Administration method: Proxy-report questionnaire • Purpose and use: Documents child's status, planning intervention, and document intervention progress	b1, d1, d2, and d7	[31] V (+), R (+)

2.	Evaluation Tool for Children's Handwriting (ETCH) [35, 42, 44]	Amundson [36], occupational therapy	United States	Assesses a child's legibility and speed of handwriting and the child's pencil management related to writing tasks commonly done in a classroom setting	• Approach: Criterion-referenced • Administration method: Performance-based and observation-based • Purpose and use: Documents child's status, planning intervention, and document intervention progress	d1 and d4	[42] V (+), R (+) [44] V (+), R (+) [35] V (−), R−)

3.	Beery-Buktenica Developmental Test of Visual-Motor Integration (VMI) [25, 35, 45, 54, 83]	Beery and Beery [26], psychologists	United States	To determine whether a child demonstrates age-appropriate visual–motor integration skills	• Approach: Norm-referenced • Administration method: Performance-based • Purpose and use: Screening and documents child's status	b1, b2, d1, and d4	[35] V (−), R (−) [45] V (+), R (+) [54] V (+), R (+) [83] V (+), R (+) [25] V (+), R (+)

4.	Minnesota Handwriting Assessment (MHA) [37, 42, 45, 55, 65, 75, 76]	Reisman [38], occupational therapy	United States	To analyze handwriting skills with first and second-grade students	• Approach: Norm-referenced • Administration method: Performance-based • Purpose and use: Screening and documents child's status	d1 and d4	[37] V (−), R (+) [45] V (+), R (+) [55] V (+), R (+) [65] V (+), R (+) [76] V (+), R (+) [75] V (+), R (+) [42] V (−), R (+)

5.	Developmental Test of Visual Perception-2 (DTVP-2) [25, 39]	Hammill et al. [27], psychologist and occupational therapy	United States	To assess visual–perceptual deficits in children and yield scores for both visual perception (no motor response) and visual–motor integration ability	• Approach: Norm-referenced • Administration method: Performance-based • Purpose and use: Screening, documents child's status, and planning intervention	b1, d1, and d4	[39] V (+), R (+) [25] V (−), R (+)

6.	Test of Visual Perceptual Skills (nonmotor) Revised (TVPS-R) [39]	Gardner [40], psychologist	United States	To measure visual–perceptual skills in seven areas: Visual discrimination, visual memory, visual–spatial relationships, visual form constancy, visual sequential memory, visual figure-ground, and visual closure	• Approach: Norm-referenced • Administration method: Performance-based • Purpose and use: Screening, documents child's status, and planning intervention	b1, d1, and d2	[39] V (+), R (+)

7.	Jordan Left-Right Reversal Test (JLRRT) [39]	Jordan [41], psychologist	United States	To measure visual reversals in children ages 5–12	• Approach: Norm-referenced • Administration method: Performance-based • Purpose and use: Screening and documents child's status	b1, b2, b7, d1, and d4	[39] V (−), R (−)

8.	Get Ready to Learn Supplemental Data Sheet [46]	Buckley-Reen [47], occupational therapy	United States	The aim is to record the special education teachers' weekly perceived impression of students' behavioral changes in the frequency and/or intensity of the four dependent variables, which are level of independence, attention, transition, and self-regulation	• Approach: Criterion-referenced • Administration method: Proxy-report questionnaire • Purpose and use: Document intervention progress	b1 and d4	[46] V (−), R (−)

9.	The Print Tool [52]	Olsen and Knapton [53], occupational therapy	United States	To evaluate eight different writing components: Memory, orientation, placement, size, start, sequence, control, and spacing. This is part of the Handwriting Without Tears (HWT) program	• Approach: Criterion-referenced • Administration method: Performance-based • Purpose and use: Documents child's status, planning intervention, and document intervention progress	d1 and d4	[52] V (−), R (−)

10.	Canadian Occupational Performance Measure (COPM) [55]	Law et al. [56], occupational therapy	Canada	Outcome measure designed to detect change in a client's perception of performance and satisfaction in self-identified problem areas	• Approach: Ipsative assessment • Administration method: Interview-based, self-report questionnaire • Purpose and use: Planning intervention and document intervention progress	d2, d4, d5, d7, and d9	[55] V (+), R (+)

11.	Loewenstein Occupational Therapy Cognitive Assessment (LOTCA) [57]	Itzkovich et al. [58], occupational therapy	Israel	To measure basic cognitive skills to perform ADLs/IADLs, including orientation, visual perceptual/psychomotor abilities, problem-solving skills, and thinking operations	• Approach: Criterion-referenced • Administration method: Performance-based and observation-based • Purpose and use: Documents child's status and planning intervention	b1 and d1	[57] V (+), R (+)

12.	Here's How I Write: A Child's Self-Assessment (HHIW) [59]	Goldstand et al. [60], occupational therapy	United States	Handwriting self-assessment to evaluate handwriting performance by children	• Approach: Ipsative assessment • Administration method: Self-report questionnaire • Purpose and use: Planning intervention and document intervention progress	d1 and d4	[59] V (+), R (−)

13.	BHK, Concise Assessment Method for Children's Handwriting [63]	Charles et al. [64], psychologist	France	To diagnose disturbances of writing in children aged 6–10 years. Evaluation based on letter size, left margin widening, poor word alignment, insufficient word spacing, acute turns in connecting letters or too long joining (chaotic writing), irregularities in joining strokes, collision of letters, inconsistent letter size, incorrect relative height of letters, letter distortion, ambiguous letter forms, correction of letter forms, and unsteady writing trace	• Approach: Norm-referenced • Administration method: Performance-based • Purpose and use: Screening and documents child's status	b1, d1, and d4	[63] V (+), R (−)

14.	Persian Handwriting Assessment Tool (PHAT) [68, 77, 81]	Havaei et al. [68], occupational therapy	Iran	To evaluate handwriting components (legibility and speed) and two major handwriting domains (near-point copying and dictation)	• Approach: Norm-referenced • Administration method: Performance-based • Purpose and use: Screening and documents child's status	b1, d1, and d4	[68] V (+), R (−) [77] V (+), R (+) [81] V (−), R (−)

15.	School Interim Competency of Performance Skill Battery Scale (SICPSBS) [69]	Nobahar et al. [69], occupational therapy	Iran	To evaluate the school readiness of students aged 5–7 years old	• Approach: Norm-referenced • Administration method: Proxy-report questionnaire • Purpose and use: Documents child's status and planning intervention	b1, b7, d1, d3, d4, and d7	[69] V (+), R (−)

16.	The Dynamic Occupational Therapy Cognitive Assessment for Children (DOTCA-Ch) [70]	Katz et al. [71], occupational therapy	Israel	To evaluate the cognitive performance of children aged 6–12 years old, allowing the identification of potential and limitations in the primary cognitive areas, related to function, as well as their short-term memory performance	• Approach: Criterion-referenced • Administration method: Performance-based • Purpose and use: Documents child's status and planning intervention	b1, b2, b4, b7, and d1	[70] V (−), R (+)

17.	Goal Attainment Scaling Scores (GAS) [72, 85]	Kiresuk and Sherman [73], psychologists	United States	To measure behavioral changes in small increments	• Approach: Ipsative assessment • Administration method: Observation-based, interview-based, self-report questionnaire, and proxy-report questionnaire • Purpose and use: Planning intervention and document intervention progress	d1, d2, d3, d4, d5, d6, and d7	[72] V (−), R (−) [85] V (−), R (+)

18.	The Perceive, Recall, Plan, and Perform (PRPP) [72]	Chapparo and Ranka [74], occupational therapy	Australia	Observational measurement of the way children uses cognitive information strategically in everyday performance	• Approach: Criterion-referenced • Administration method: Performance-based and observation-based • Purpose and use: Documents child's status, planning intervention, and document intervention progress	b1, d2, and d3	[72] V (+), R (+)

19.	Children's Sleep Habit Questionnaire (CSHQ) [78]	Owens et al. [79], psychologists	United States	To evaluate sleep habits of children aged 7–12 years old by questioning bedtime resistance, sleep onset delay, sleep duration, sleep anxiety, night wakings, parasomnias, sleep-disordered breathing, and daytime sleepiness	• Approach: Norm-referenced • Administration method: Proxy-report questionnaire • Purpose and use: Screening and documents child's status	b1	[78] V (+), R (+)

20.	Sensory Profile Questionnaire (SPQ) [78]	Dunn [80], psychologist	United Kingdom	Evaluate the frequency of behaviors related to sensory processing	• Approach: Norm-referenced • Administration method: Proxy-report questionnaire • Purpose and use: Screening and documents child's status	b1 and b2	[78] V (−), R (+)

21.	School Function Assessment (SFA) [33, 84]	Coster et al. [34], occupational therapy	United States	To evaluate and monitor a student's participation, support needs, and performance of functional (nonacademic) tasks and activities that affect the academic and social aspects of the school environment	• Approach: Criterion-referenced • Administration method: Observation-based and proxy-report questionnaire • Purpose and use: Documents child's status, planning intervention, and document intervention progress	b1, b2, d1, d4, d7, d8, e3, and e4	[84] V (+), R (+) [33] V (−), R (−)

22.	Short Child Occupational Profile (SCOPE) [85]	Bowyer et al. [86], occupational therapy	United States	To assess factors that represent the MOHO concepts of skills, volition, habituation, and the environment, regardless of the child's symptoms, diagnosis, age, or the treatment setting and to support occupation-focused intervention	• Approach: Ipsative assessment • Administration method: Observation-based and proxy-report questionnaire • Purpose and use: Planning intervention and document intervention progress	b1, d3, d5, d7, d8, e3, and e4	[85] V (−), R (−)

23.	Behavior Assessment System for Children, Third Edition (BASC–3) [85]	Reynolds and Kamphaus [87], psychologists	United States	Analyze the child's behavior from three perspectives—self, teacher, and parent—using a comprehensive set of rating scales and forms to help understand the behaviors and emotions of children and adolescents	• Approach: Norm-referenced • Administration method: Self-report questionnaire, proxy-report questionnaire, and observation-based • Purpose and use: Screening and documents child's status	b1, d7, d8, e3, and e4	[85] V (−), R (−)

24.	Motor Assessment Battery for Children 2nd Edition (MABC-2) [88]	Henderson et al. [92], psychologists	United Kingdom	Measurement of gross and fine motor coordination difficulties in children and young adults aged 3–25 years	• Approach: Norm-referenced • Administration method: Performance-based and proxy-report questionnaire • Purpose and use: Screening, documents child's status, planning intervention, and document intervention progress	b7 and d4	[88] V (−), R (+)

25.	Colored Progressive Matrices Test (CPM) [88, 98]	Raven et al. [89], psychologists	United Kingdom	Evaluate nonverbal intelligence among children 5–11 years old	• Approach: Norm-referenced • Administration method: Performance-based • Purpose and use: Screening and documents child's status	b1 and d1	[88] V (−), R (+) [98] V (−), R (+)

26.	Finger Succession (FS) [88, 98]	Berninger and Rutberg [91], educational psychologist	United States	Examines fine motor sequencing and its impact on learning-related tasks	• Approach: Norm-referenced • Administration method: Performance-based • Purpose and use: Screening and documents child's status	d4	[88] V (−), R (−) [98] V (−), R (+)

27.	Children's Color Trails Test (CCTT) [88, 98]	Llorente et al. [93], neuropsychologist	United States	Examine sustained visual attention among 8–16-year-old children and adolescents	• Approach: Norm-referenced • Administration method: Performance-based • Purpose and use: Screening and documents child's status	b1 and d1	[88] V (+), R (+) [98] V (+), R (+)

28.	Arabic Handwriting Assessment (A-HAT) [88]	Salameh-Matar et al. [94], occupational therapy	Israel	Evaluates the Arabic handwriting of elementary school students, including a paragraph copying task and to writing-to-dictation task	• Approach: Norm-referenced • Administration method: Performance-based • Purpose and use: Screening and documents child's status	b1, d1, and d4	[88] V (−), R (+)

29.	Arabic Keyboarding Assessment (A-KBAT) [88]	Author concealed	Israel	Evaluate the Arabic keyboarding performance of students in fourth and fifth grades. It includes a 5-min copying task and a 3-min writing-to-dictation task	• Approach: Norm-referenced • Administration method: Performance-based • Purpose and use: Screening and documents child's status	b1, d1, and d4	[88] V (+), R (−)

30.	Primary Spelling Inventory (PSI) [96]	Bear et al. [97], educator	United States	Measures the spelling ability of students in kindergarten through third grade and consists of 26 words that are listed in order by difficulty	• Approach: Criterion-referenced • Administration method: Performance-based • Purpose and use: Documents child's status and planning intervention	b1, d1	[96] V (−), R (−)

31.	Aleph-Taph [98]	Shany et al. [99], educator	Israel	Assesses reading, verbal memory, and linguistic skills in Hebrew	• Approach: Norm-referenced • Administration method: Performance-based • Purpose and use: Screening, documents child's status, and planning intervention	b1 and d1	[98] V (−), R (−)

32.	Purdue Pegboard Test (PPT) [98]	Tiffin [100], psychologist	United States	To evaluate gross movements of the fingers, hands, and arms and fine fingertip dexterity necessary in assembly tasks	• Approach: Norm-referenced • Administration method: Performance-based • Purpose and use: Screening, documents child's status, and document intervention progress	b7 and d4	[98] V (−), R (+)

33.	Hebrew Keyboarding Assessment (H-KBAT) [98]	Weintraub et al. [101], occupational therapy	Israel	Evaluate the Hebrew keyboarding performance of students in fourth to sixth grades	• Approach: Criterion-referenced • Administration method: Performance-based • Purpose and use: Documents child's status and document intervention progress	b1, d1, and d4	[98] V (+), R (+)

34.	Korea-Child Behavior Checklist (K-CBCL) [102]	Oh et al. [103], pediatric psychiatric	South Korea	Standardized child and adolescent behavior assessment tool that translates the Child's Behavior Checklist developed	• Approach: Norm-referenced • Administration method: Proxy-report questionnaire • Purpose and use: Screening, documents child's status, and document intervention progress	b1, d1, d7, and d8	[102] V (−), R (+)

35.	School Life Adjustment Scale [102]	Jo and Doh (2018), educational psychologist	South Korea	Evaluated school life adjustment of children in special classes, including class attitude, friendship, positive personal behavior, and school rules, with a total of 20 questions	• Approach: Norm-referenced • Administration method: Proxy-report questionnaire • Purpose and use: Documents child's status and document intervention progress	d7, d8, and d9	[102] V (−), R (−)

36.	Sensory Processing Assessment Tool for Schools [102]	Cho [105], occupational therapy	Korea	Evaluate behaviors related to sensory processing difficulties in the school life of school-age children with detailed areas consisting of tactile processing, movement processing, visual processing, auditory processing, olfactory processing, and multisensory processing	• Approach: Norm-referenced • Administration method: Proxy-report questionnaire and observation-based • Purpose and use: Screening and documents child's status	b1	[102] V (−), R (−)

37.	Bruininks-Oseretsky Test of Motor Proficiency 2 Short Form [102]	Bruininks and Bruininks [106], psychologist	United States	To provide a comprehensive overview of fine and gross motor skills in children and young adults within the school age range	• Approach: Norm-referenced • Administration method: Performance-based and observation-based • Purpose and use: Screening and documents child's status	b1, b7, d1, and d4	[102] V (−), R (−)

38.	Diagnostic Reading Test in Arabic for Elementary School Students, 2nd−6th grades [88]	Taha [90], Arabic language and literacy educator	Egypt	Designed to assess children with behavior problems in school settings used by teachers, parents, or child raters	• Approach: Norm-referenced • Administration method: Performance-based • Purpose and use: Documents child's status	b1, d1, and d2	[88] V (+), R (−)

39.	Child and Adolescent Trial for Cardiovascular Health: Health Behavior Questionnaires (HBQ) [48]	Perry et al., [49], public health, epidemiologists, cardiologists, behavioral scientists, nutritionists, physical educators, and psychologists	United States	Evaluates specific health-related behaviors such as diet, physical activity, and cardiovascular health habits based on public health guidelines for children	• Approach: Criterion-referenced • Administration method: Self-report questionnaire • Purpose and use: Documents child's status and document intervention progress	b4, d5, and d9	[48] V (−), R (−)

40.	The Physical Activity Questionnaire (elementary school) [48]	Kowalski et al. [50], kinesiology researchers	Canada	Assess general levels of physical activity in school-aged children	• Approach: Criterion-referenced • Administration method: Self-report questionnaire • Purpose and use: Documents child's status and document intervention progress	d4 and d9	[48] V (−), R (**+**)

41.	Test of Playfulness (ToP) [61]	Skard and Bundy [62], occupational therapy	United States	Evaluate a child's playfulness by measuring four key components: Perception of control, freedom from reality constraints, source of motivation, and ability to give and read social cues	• Approach: Criterion-referenced • Administration method: Observation-based • Purpose and use: Documents child's status and planning intervention	d7, d8, and d9	[61] V (+), R (+)

42.	Kids' Imaging Ability Questionnaire (KIAQ) [65]	Kwekkeboom et al. [66], nurse	United States	Assess a child's ability to form mental images, particularly about pain and coping strategies	• Approach: Criterion-referenced • Administration method: Self-report questionnaire • Purpose and use: Screening and documents child's status	b1	[65] V (+), R (+)

43.	Peer Social Maturity Scale (PSMAT) [82]	Fink et al. [136], psychologists	Australia	Measure children's social maturity within classroom peer groups	• Approach: Norm-referenced • Administration method: Proxy-report questionnaire • Purpose and use: Screening and documents child's status	d2 and d7	[82] V (−), R (+)

44.	Handwriting Proficiency Screening Questionnaire for Children (HPSQ-C) Spanish version [107]	Romero-Ayuso et al. [107], occupational therapy	Spain	Identify handwriting proficiency issues early, enabling appropriate interventions	• Approach: Criterion-referenced • Administration method: Performance-based and self-report questionnaire • Purpose and use: Screening and documents child's status	b1 and d1	[107] V (+), R (−)

45.	The EASI [108]	Mailloux et al. [109], occupational therapy	United States	Assesses key sensory processing areas, including sensory perception, postural control, motor integration, praxis, and sensory reactivity in children aged 3–12 years	• Approach: Norm-referenced • Administration method: Performance-based and observation-based • Purpose and use: Documents child's status and planning intervention	b1, b2, b7, b8, d1, d4, d7, and d8,	[108] V (+), R (+)

46.	Handwriting Legibility Scale (HLS) [108]	Barnett et al. [110], psychologists	United Kingdom	Evaluate handwriting legibility in children and adolescents based on letter formation, size, spacing, alignment, and consistency	• Approach: Criterion-referenced • Administration method: Performance-based and observation-based • Purpose and use: Screening and documents child's status	d1 and d4	[108] V (−), R (+)

47.	Activity Clock [111]	Kugel et al. [112], occupational therapy	Canada	Exploring the time use of children aged 8–9 years old	• Approach: Ipsative assessment • Administration method: Self-report questionnaire and proxy-report questionnaire • Purpose and use: Planning intervention and document intervention progress	d2 and d5	[111] V (+), R (+)

48.	Physical and Social Well-Being Ladders [111]	Thompson and Aked [113], well-being research, policy development, and consulting	United Kingdom	Determine self-perception of physical and social well-being	• Approach: Ipsative assessment • Administration method: Observation-based and self-report questionnaire • Purpose and use: Documents child's status and document intervention progress	e4	[111] V (−), R (−)

49.	Woodcock–Johnson III Tests of Achievement (WJIII) [42, 44]	Woodcock et al., [43], psychologists	United States	Measuring individual academic performance in reading, mathematics, written language, and academic knowledge	• Approach: Norm-referenced • Administration method: Performance-based • Purpose and use: Documents child's status and planning intervention	b1, b2, d1, d2, and d3	([42]) V (−), R (+) [44] V (−), R (+)

50	Chinese Handwriting Analysis System (CHAS) [51]	Li-Tsang et al. [137], occupational therapy	China	To assess and analyze Chinese handwriting performance in primary school students	• Approach: Norm-referenced • Administration method: Performance-based • Purpose and use: Documents child's status and planning intervention	b1, b7, and d1	[51] V (+), R (+)

51	Behavior Rating Inventory of Executive Functioning (BRIEF) [29]	Gioia et al. [30], psychologists	United States	To assess the behavioral manifestations of executive function (EF) skills in the individual's everyday environment (e.g., at home and at school)	• Approach: Norm-referenced • Administration method: Self-report questionnaire and proxy-report questionnaire • Purpose and use: Screening, documents child's status, and planning intervention	b1, d1, and d2	[29] V (+), R (+)

52.	Bruininks-Oseretsky Test of Motor Proficiency [25]	Bruininks [28], psychologist	United States	To measure of fine and gross motor skills in children and young adults (typically ages 4–21)	• Approach: Norm-referenced • Administration method: Performance-based • Purpose and use: Documents child's status and planning intervention	b1, b7, d1, and d4	[25] V (−), R (−)

*Note:* ICF chapters' code information is described in Table S1 (see supporting information). The psychometric tool includes V: validity and R: reliability; (+) mentioned in the articles, (−) not mentioned in the articles.

**Table 3:** Summary of measurement tools by ICF classification.

**ICF component**	**Number of measurements**	**Percentage**	**Example tools**
Body functions	5	9.6%	Children's Color Trails Test (CCTT)
Activities and participation	12	23.1%	Finger Succession (FS) and Canadian Occupational Performance Measure (COPM)
Environmental factors	1	1.9%	Physical and Social Well-Being Ladders
Body functions + activities and participation	31	59.6%	Beery-Buktenica Developmental Test of Visual-Motor Integration (VMI) and Teacher Skill streaming Checklist
Body functions + activities and participation + environmental factors	3	5.8%	School Function Assessment (SFA) and Short Child Occupational Profile (SCOPE)

## Supporting Information

Supporting Information Additional supporting information can be found online in the Supporting Information section. We provide detailed table information about the classification component coded with the letters b, s, d, and e, followed by a numeric code of the chapter number from the International Classification of Functioning, Disability, and Health—Child and Youth Version (ICF-CY).
