# Supplementary material for: Measurement Tools in Occupational Therapy Practice Primary School Setting: A Scoping Review
Source: Occup Ther Int. 2025 Dec 3;2025:2679849. doi: 10.1155/oti/2679849 (PMC12695414; doi:10.1155/oti/2679849)
Supplement: Supporting Information — Additional supporting information can be found online in the Supporting Information section. We provide detailed table information about the classification component coded with the letters b, s, d, and e, followed by a numeric code of the chapter number from the International Classification of Functioning, Disability, and Health—Child and Youth Version (ICF-CY). [file 2679849.f1.docx]

Table A: List of ICF components and chapters

| International classification of functioning, disability, and health framework for occupational therapy | | | | | | | | |
| --- | --- | --- | --- | --- | --- | --- | --- | --- |
| Occupational Therapy Classification | Performance components | | occupational performance | | occupational performance, role competence | | environmental factors | |
| ICF Dimension | Chapters | Body functions (b) | Chapters | Body structures (s) | Chapters | Activities and participation (d) | Chapters | Environmental factors (e) |
|  | b1 | Mental functions | s1 | Structures of the Nervous System | d1 | Learning and applying knowledge | e1 | Product and technology |
|  | b2 | Sensory functions and pain | s2 | The eye, ear and related structures | d2 | General tasks and demands | e2 | Natural environment and human-made changes to the environment |
|  | b3 | Voice and speech functions | s3 | Structures involved in voice and speech | d3 | Communication | e3 | Support and relationships |
|  | b4 | Functions of the cardiovascular, haematological, immunological and respiratory systems | s4 | Structures of the cardiovascular, immunological and respiratory | d4 | Mobility | e4 | Attitudes |
|  | b5 | Functions of the digestive, metabolic and endocrine systems | s5 | Structures related to the digestive, metabolic and endocrine systems | d5 | Self-care | e5 | Services, systems and policies |
|  | b6 | Genitourinary and reproductive functions | s6 | Structures related to the genitourinary and reproductive systems | d6 | Domestic life |  |  |
|  | b7 | Neuromusculoskeletal and movement-related functions | s7 | structures related to movement | d7 | Interpersonal interactions and relationships |  |  |
|  | b8 | Functions of the skin and related structures | s8 | skin and related structures | d8 | Major life areas |  |  |
|  |  |  |  |  | d9 | Community, social and civic life |  |  |
